# An Environmentally Compatible and Less Costly (Greener) Microwave Digestion Method of Bone Samples Using Dilute Nitric Acid for Analysis by ICP-MS

**DOI:** 10.3390/molecules29235517

**Published:** 2024-11-22

**Authors:** Derek D. Bussan, Forrest H. Nielsen, Chris Douvris, Brett Kelzenberg, Allison Grimestad, Jay J. Cao

**Affiliations:** 1 United States Department of Agriculture—Agricultural Research Service (USDA-ARS), Grand Forks Human Nutrition Research Center, Grand Forks, ND 58203, USA; forrest.nielsen@usda.gov (F.H.N.); brett.kelzenberg@usda.gov (B.K.); allison.grimestad@usda.gov (A.G.); jay.cao@usda.gov (J.J.C.); 2 Theobald Science Center, Department of Biological and Chemical Sciences, New York Institute of Technology, Old Westbury, NY 11568, USA; cdouvris@nyit.edu

**Keywords:** ICP-MS, microwave assisted digestion, elemental analysis, green method

## Abstract

An environmentally compatible and less costly (greener) analytical method for the digestion of bone meal samples using microwave-assisted dilute nitric acid (HNO_3_) was developed and optimized. The method, employing a mixture of 1 mL concentrated HNO_3_ and 4 mL of deionized water, offered a comparable performance to the conventional method using 5 mL of concentrated HNO_3_. The accuracy of the method was validated by using certified reference material NIST 1486 (Bone Meal); percentage recoveries were within ±15% for all eight certified elements. Statistical analysis revealed no significant differences (*p* > 0.05) in percentage recoveries between the green and conventional methods for all elements except calcium. The greenness of the developed method was evaluated by using the analytical Eco-Scale, achieving a score of 87, categorizing it as an “excellent green analysis” method. This research highlights the potential for adopting greener practices in trace element analysis that reduce the environmental impact and safety risks associated with concentrated acids.

## 1. Introduction

Human safety, accurate and reproducible results, and lowering both resource consumption and costs are high priorities in laboratory science. In many modern analytical laboratories, reducing or eliminating the generation or use of hazardous substances causing negative environmental impacts (green science) is a priority issue [1,2]. Microwave wet digestion (MWD) of samples for trace element analysis makes this issue more achievable because modern microwave wet digestion systems (MWDSs) remove much of the potential for human error and harm while providing a controlled, reproducible process. Improving MWD could minimize analyte loss and contamination, enhance human and environmental safety, and save resources and time.

Using MWD aids in the digestion of organic and inorganic samples for trace element analyses by inductively coupled plasma mass spectrometry (ICP-MS). Using a MWDS demands the use of the correct methods to achieve samples needed to obtain accurate results. The misuse of a MWDS and acid concentration often results in errors in filtering the digestion solution, incomplete digestion, and higher lab time costs [3].

A vast amount of research has used MWD and ICP-MS technology to determine the trace element content of food and drink, microplastics, cannabis, and consumer materials [4,5,6,7]. With the evolution of ICP-MS, it is imperative that the MWD method similarly advances in productivity and efficiency, particularly in the use of diluted acids [8,9].

Dilution has been used to avoid the dangers of utilizing high concentrations of HNO_3_ in the digestion of samples [10]. However, using diluted HNO_3_ may result in incomplete digestion [11]. One method to overcome this issue is to add hydrogen peroxide to HNO_3_ to increase oxidative power [8,12,13]. Furthermore, some samples digested using a decreased amount of HNO_3_ have given mixed results in residual carbon content in the final digestate [14]. In one study, samples containing high levels of crude protein had a low carbon residual content, but in samples with a lower crude protein content, the residual carbon content was higher [9].

Cost effectiveness also needs to be considered when using chemicals in the laboratory. Using high-purity acids to digest samples for ICP-MS analysis is expensive. A common practice is to use Optima Grade HNO_3_, which is the highest purity and most expensive form of HNO_3_ [15,16,17]. There is a significant price difference between Optima Grade HNO_3_ and the next highest grade which is Trace Metal Grade.

Because of the effectiveness and efficiency of MWD, which has the potential to be modified to a safer, greener method, the objective of this study was to determine whether dilute HNO_3_ acid could be used in MWD before ICP analysis with a maintenance of adequate percentage recovery and accurate results [18,19]. A method for the chemical digestion of bone with dilute nitric acid was developed and was tested to see if it was optimal for ICP-MS analysis.

## 2. Materials and Methods

### 2.1. Instrumentation

Sample digestion was performed by a Single Mode Cavity (SMC) microwave (Blade, CEM, Matthews, NC, USA) equipped with an auto sampler that can handle 24 high-purity quartz vessels with snap-on caps. The microwave can operate at a temperature and pressure of up to 300 °C and 700 psi (corresponding to 4.8 MPa), respectively. The blade MWDS continuously stirs each individual sample during the digestion process. Instrumental settings used to digest samples and run blanks are given in Supplementary Materials Table S1. Once digested, samples were allowed to cool before being diluted for trace element analysis.

The determination of certified elements in the standard reference material (SRM), which were phosphorus (P), magnesium Mg), iron (Fe), zinc (Zn), potassium (K), calcium (Ca), strontium (Sr), and lead (Pb), was performed using ICP-MS (Nexion 5000, Perkin Elmer, Shelton, CT, USA) under the following operational conditions: nebulizer gas flow 0.92 L min^−1^, auxiliary gas flow 1.2 L min^−1^, plasma gas flow 16 L min^−1^, radio-frequency power of 1600 W, quadrupole ion deflector (QID) fixed power −12 V, hyperskimmer park voltage 3.5 V, OmniRing Park Voltage −195 V, inner target lens voltage 1.5 V, outer target lens voltage −3.5 V, deflector exit voltage −8 V, differential aperture voltage −5 V, cell rod offset −34 V, analog stage voltage −1750 V, pulse stage voltage 1100. Q1 parameters; Q1 AC rod offset −5 V, Q1 Rod offset 0 V, cell entrance voltage −10 V, cell exit voltage −15 V. Q3 parameters; Q1 AC rod offset −5.5 V, Q1 Rod offset −19 V, cell entrance voltage −6 V, cell exit voltage −3 V. MS/MS mode parameters Q1 AC rod offset −7 V, Q1 rod offset 0 V, cell entrance voltage −1 V, and cell exit voltage 0 V. Tables S2–S9 contain the operating conditions for each of the 8 elements analyzed. Scandium was used as an internal standard. Argon with a spectral purity of 99% (American Welding and Gas, West Fargo, ND, USA) was used for plasma, nebulization, and auxiliary gas. Measurements were performed using the focusing analysis mode. Calibration standards, deionized water blanks, and scandium as an internal standard were utilized to monitor the drift of the instrument during the analysis. Deionized water blanks were used to identify any loss or cross-contamination.

### 2.2. Certified Reference Material, Reagents and Standards

NIST 1486 (bone meal) obtained from the National Institute of Standards and Technology (Gaithersburg, MD, USA) was used to evaluate the accuracy and precision of the conceived method. For digestion, 20 mg of bone meal was added to each quartz vessel containing either 5 mL Optima™ HNO_3_ (Fisher Scientific, Hampton, NH, USA) or a mixture of 4 mL deionized water with a resistance of 18 MΩ cm^−1^ (Milli-Q^®^ System Millipore, Sigma Aldritch, Boston, MA, USA) and 1 mL Optima™ Grade HNO_3_. The Optima™ Grade HNO_3_ used in this study is specifically designed for ICP-MS and high-resolution ICP-MS analysis, with certified evaluation for 65 metals at low-level ppt concentrations. The reagent is manufactured, purified, and packaged in a Class 1000 clean room, and is contained in specially manufactured and cleaned FEP fluoropolymer bottles to maintain product integrity and eliminate metal leaching that can occur with glass containers. The product carries a three-year shelf life from the date of manufacture, as documented on the Certificate of Analysis. Three method blanks of each solution without bone meal were also prepared simultaneously with each digestion run. Single element ICP-MS calibration standards purchased from AccuStandard, Inc. (New Haven, CT, USA) were used to prepare the calibration curves. Standard calibration solutions were prepared in the range of 0.1 ng L^−1^ to 300 ng L^−1^ for (Fe, Zn, Sr, Pb), 10 ng L^−1^ to 200 ng L^−1^ for K, 1 ng L^−1^ to 220 ng L^−1^ for Mg, 5 mg L^−1^ to 40 mg L^−1^ for Ca, 3 mg L^−1^ to 40 mg L^−1^ for P. The solutions were prepared by diluting the stock solutions with 0.5% (*w*/*w*) HNO_3_.

### 2.3. Analytical Quality Control and Statistical Analysis

Matrix effects were evaluated for the conceived method, and quality performance parameters, including linearity, accuracy, precision and limit of detection (LOD). The linearity (correlation coefficient) was obtained from the external calibration curve of the elements by using the least-squares linear regression method. All calibration curves demonstrated excellent linearity with correlation coefficients (r^2^) > 0.995. The accuracy (percentage recovery) was calculated by comparing the determined values with certified values of CRM, and precision (repeatability) was evaluated by the relative standard deviation (percent RSD) of the five replicates ran for each method. The limit of detection (LOD) and limit of quantification (LOQ) were calculated as 3 and 10 times the standard deviation of the method blank analytical signals (*n* = 3), respectively. The significant differences (*p* < 0.05) between the two methods were carried out with One-way ANOVA (JMP, version 17, SAS Institute, Inc., Cary, NC, USA).

## 3. Results and Discussion

The selection of the appropriate acid for microwave-assisted acid digestion of materials prior to ICP mineral analysis is critical, as this has become a method of choice for the digestion of a wide range of matrices, including those derived from food sources. Among the various oxidizing agents employed in acid digestion protocols, concentrated nitric acid is most commonly utilized because it is available in high purity and has extensive oxidative capacity [12]. Moreover, our modified digestion method using 4 mL water and 1 mL nitric acid represents a greener analytical approach compared to traditional methods that rely on larger volumes of pure nitric acid. Previous studies have reported using significantly higher amounts of concentrated nitric acid—15 mL for lead determination in bones (Yoon et al., 2003) and 8 mL for calcium and phosphorus analysis (Fleischer et al., 2014) [20,21]. By reducing nitric acid consumption by 80–93% while maintaining analytical performance, our method aligns with green chemistry principles. Alternative reported approaches to reduce this acidic load include the use of diluted HNO_3_ in conjunction with hydrogen peroxide as an auxiliary oxidizing agent [8,12,13], and high-temperature and high-pressure systems in combination with diluted HNO_3_ [22]. The usage of HCl was not necessary for this digestion protocol, as complete dissolution was achieved and further validated by the excellent percentage recoveries achieved with the standard reference materials. The conceived new green method depicts an effort to reduce acidity that results in more environmentally benign digests. The digestion procedure for the NIST-1486 bone meal samples was based solely on the use of HNO_3_ and water without the use of an auxiliary oxidizing reagent. After evaluating various dilutions of concentrated nitric acid, we determined the optimal composition to be 1 mL of concentrated HNO_3_ combined with 4 mL of H_2_O.

Following microwave digestion with the diluted acid, ICP-MS analysis was conducted for the eight elements that were certified for the CRM NIST 1486 (Bone Meal). The LOD and LOQ for these elements are presented in Table 1. There was no residue after the digestion.

Table 2 presents the percentage recovery values using the new green method; all were within ±15% of the known concentrations in the CRM. This excellent agreement is a verification of the accuracy of the new green method.

Table 3 shows the percentage recoveries using the new reported green digestion protocol based on diluted nitric acid compared with those obtained with the conventional concentrated nitric acid digestion protocol using 5 mL of concentrated nitric acid. For all the elements except Ca, there is no statistically significant difference (*p* > 0.05) in the percentage recoveries between the green and conventional method. Statistically, the recovery of calcium (Ca) was significantly different (*p* = 0.01), which indicates a slight variance in effectiveness between the methods. Despite this variance, the overall results suggest that the green method has a comparable level of performance to the conventional method, which shows that it is a suitable, safer, and more sustainable alternative to be used in trace element analyses. The data precision is presented in Table 2, where each measured value is accompanied by its standard deviation (±), which quantifies the measurement uncertainty and reproducibility of our analytical method. This notation, following standard scientific practice, represents one standard deviation from the mean of replicate measurements.

The higher values observed for calcium (Ca) and phosphorus (P) directly correlate with their higher concentrations in the reference materials used in this study, as these elements are the major constituents of bone tissue. This proportional relationship between concentration and measured values is expected and validates our analytical method. The data precision is presented in Table 2, where each measured value is accompanied by its standard deviation (±), which quantifies the measurement uncertainty and reproducibility of our analytical method. This notation, following standard scientific practice, represents one standard deviation from the mean of replicate measurements.

The greenness of the analytical procedures using the conceived method was assessed by using the analytical Eco-Scale [18]. According to this scale, the ideal score is 100; penalty points are given for each parameter that is different from the ideal score, resulting in a lower total score. Analytical methods with Eco-Scale scores higher than 50 and 75 are considered “acceptable green analysis” and “excellent green analysis”, respectively [18].

For the conceived, five penalty points were deducted because of the use of HNO_3_ and microwave-assisted heating [23]. An additional five penalty points were assessed because of the use of multi-elemental solutions for calibration and plasma-based techniques, and three penalty points for waste generation. Thus, the final score of 87 for the conceived method categorized it as an “excellent green analysis” method.

## 4. Conclusions

A green analytical method for the digestion of samples for ICP-MS analyses was developed and optimized. The method composed of 1 mL of concentrated HNO_3_ and 4 mL of deionized water offered comparable results to the conventional method that employs only concentrated HNO_3_. The accuracy of the new green method was validated through the analysis of certified reference material NIST 1486 (Bone Meal); percentage recoveries were within ±15% for all eight certified elements (P, Mg, Fe, Zn, K, Ca, Sr, and Pb). Statistical analysis revealed no significant differences (*p* > 0.05) in the percentage recoveries between the new green and the conventional method, with the exception of Ca. Despite this slight Ca variance, the overall results support the validity of the green method as a safer and more sustainable alternative to conventional digestion practices without compromising analytical quality. An analysis of the new green method using the analytical Eco-Scale gave a final score of 87, which corresponds to an “excellent green analysis” rating, highlighting its environmental friendliness. Overall, the green analytical method not only reduces the environmental impact and safety risks associated with the use of concentrated acids, but also offers a cost-effective alternative to traditional sample preparation techniques. The implications of this research may extend beyond the specific analysis of bone meal samples to other sample matrices. Future work should involve testing a broader range of environmental and biological samples.

## Figures and Tables

**Table 1:** Limit of detection (LOD) and limit of quantification (LOQ).

Analyte	LOD	LOQ
P	0.08 mg L^−1^	0.26 mg L^−1^
Mg	0.76 µg L^−1^	2.52 µg L^−1^
Fe	0.05 µg L^−1^	0.16 µg L^−1^
Zn	0.016 µg L^−1^	0.05 µg L^−1^
K	0.13 mg L^−1^	0.44 mg L^−1^
Ca	0.02 mg L^−1^	0.07 mg L^−1^
Sr	0.006 mg L^−1^	0.02 mg L^−1^
Pb	0.11 µg L^−1^	0.36 µg L^−1^

**Table 2:** Measured values and % recovery in NIST 1486 bone meal samples (*n* = 5) microwaved digested in 1 mL of HNO_3_/4 mL of H_2_O.

Analyte	Reference Value (mg kg^−1^)	Measured Value (mg kg^−1^)	Percentage Recovery (%)
P	123,000 ± 1900	125,952 ± 9594	102.4
Mg	4660 ± 170	4119 ± 205	88.4
Fe	99 ± 8	88 ± 6	89.2
Zn	147 ± 16	141 ± 9	95.8
K	412 ± 4	383 ± 12	93.0
Ca	265,800 ± 2400	254,371 ± 6645	95.7
Sr	264 ± 7	243 ± 10	92.1
Pb	1.335 ± 0.014	1.2816 ± 0.084	96.0

**Table 3:** Measured values and % recovery in NIST 1486 bone meal samples. (*n* = 5); 5 mL of HNO_3_.

Analyte	Reference Value (mg kg^−1^)	Measured Value (mg kg^−1^)	Percentage Recovery (%)	*p*-Value
P	123,000 ± 1900	128,166 ± 6150	104.2	0.7
Mg	4660 ± 170	4450 ± 247	95.5	0.07
Fe	99 ± 8	95.4 ± 6.9	95.4	0.31
Zn	147 ± 16	131 ± 7	89.3	0.16
K	412 ± 4	396 ± 18	96.2	0.25
Ca	265,800 ± 2400	276,698 ± 14,619	104.1	0.01
Sr	264 ± 7	251 ± 15	95.0	0.42
Pb	1.335 ± 0.014	1.2709 ± 0.072	95.2	0.6

## Data Availability

Available upon request.

## Supplementary Materials

The following supporting information can be downloaded at: https://www.mdpi.com/article/10.3390/molecules29235517/s1, Table S1: BLADE MWDS Settings for Bone Method; Table S2: ^44^Ca ICP-MS Method; Table S3: ^39^K ICP-MS Method; Table S4: ^31^P ICP-MS Method; Table S5: ^24^Mg ICP-MS Method; Table S6: ^65^Zn ICP-MS Method; Table S7: ^57^Fe ICP-MS Method; Table S8: ^208^Pb ICP-MS Method; Table S9: ^88^Sr ICP-MS Method; Table S10: Blade MWDS Settings for Bone Method.
