# Supplementary material for: An Environmentally Compatible and Less Costly (Greener) Microwave Digestion Method of Bone Samples Using Dilute Nitric Acid for Analysis by ICP-MS
Source: Molecules. 2024 Nov 22;29(23):5517. doi: 10.3390/molecules29235517 (PMC11643719; doi:10.3390/molecules29235517)
Supplement: Supplementary file 1 [file molecules-29-05517-s001.zip › molecules-3324729-supplementary.pdf]

Table S1. BLADE MWDS Settings for Bone Method

Stage 1	
Temperature (°C)	220
Pressure (psi)	700
Stirring	Medium
Ramp Time	5:00 minutes
Hold Time	5:00 minutes
Vent Pressure (PSI)	
1	225
2	250
3	275
4	325
5	750
Vent Count	
1	2
2	2
3	2
4	2
5	∞
Sensors	
Temperature (°C)	42.2
I/O Board Temperature (°C)	39.7
AC Board Temperature (°C)	33.9
Pressure (psi)	0.0
Line Voltage (V)	120.0
Line Frequency (Hz)	60

Table S2. ^{44}Ca ICP-MS Method

Parameter	Operating Condition
Sweeps/Reading	10
Readings/Replicate	10
Replicates	3
Internal Standard ^{45}Sc	
Scan Mode	MS/MS
MCA Channels	1
IGM	Focusing
Dwell Time/ AMU	50
Integration Time	5000
Profile	Standard
Ammonia	0
Helium	0
Hydrogen	0
Oxygen	0
RPa	0
RPq	0.25
^{44}Ca	
Scan Mode	MS/MS
MCA Channels	1
IGM	Focusing
Dwell Time/ AMU	50
Integration Time	5000
Profile	Ammonia DRC
Ammonia	0.5
Helium	0
Hydrogen	0
Oxygen	0
RPa	0
RPq	0.45

Table S3. ^{39}K ICP-MS Method

Parameter	Operating Condition
Sweeps/Reading	5
Readings/Replicate	5
Replicates	5
Internal Standard ^{45}Sc	
Scan Mode	MS/MS
MCA Channels	1
IGM	Focusing
Dwell Time/ AMU	2
Integration Time	50
Profile	Ammonia DRC
Ammonia	0.7
Helium	0
Hydrogen	0
Oxygen	0
RPa	0
RPq	0.85
^{39}K	
Scan Mode	MS/MS
MCA Channels	1
IGM	Focusing
Dwell Time/ AMU	2
Integration Time	50
Profile	Ammonia DRC
Ammonia	0.7
Helium	0
Hydrogen	0
Oxygen	0
RPa	0
RPq	0.85

Table S4. ^{31}P ICP-MS Method

Parameter	Operating Condition
Sweeps/Reading	5
Readings/Replicate	5
Replicates	5
Internal Standard ^{45}Sc	
Scan Mode	MS/MS
MCA Channels	1
IGM	Focusing
Dwell Time/ AMU	2
Integration Time	50
Profile	Standard
Ammonia	0
Helium	0
Hydrogen	0
Oxygen	0
RPa	0
RPq	0.25
^{31}P	
Scan Mode	MS/MS
MCA Channels	1
IGM	Focusing
Dwell Time/ AMU	2
Integration Time	50
Profile	Helium KED
Ammonia	0
Helium	0.8
Hydrogen	0
Oxygen	0
RPa	0.015
RPq	0.25

Table S5. ^{24}Mg ICP-MS Method

Parameter	Operating Condition
Sweeps/Reading	10
Readings/Replicate	10
Replicates	5
Internal Standard ^{45}Sc	
Scan Mode	MS/MS
MCA Channels	1
IGM	Focusing
Dwell Time/ AMU	2
Integration Time	200
Profile	Standard
Ammonia	0
Helium	0
Hydrogen	0
Oxygen	0
RPa	0
RPq	0.25
^{24}Mg	
Scan Mode	MS/MS
MCA Channels	1
IGM	Focusing
Dwell Time/ AMU	2
Integration Time	200
Profile	Helium KED
Ammonia	0
Helium	3
Hydrogen	0
Oxygen	0
RPa	0
RPq	0.25

Table S6. ^{65}Zn ICP-MS Method

Parameter	Operating Condition
Sweeps/Reading	5
Readings/Replicate	5
Replicates	5
Internal Standard ^{45}Sc	
Scan Mode	MS/MS
MCA Channels	1
IGM	Focusing
Dwell Time/ AMU	2
Integration Time	50
Profile	Standard
Ammonia	0
Helium	0
Hydrogen	0
Oxygen	0
RPa	0
RPq	0.25
^{65}Zn	
Scan Mode	MS/MS
MCA Channels	1
IGM	Focusing
Dwell Time/ AMU	2
Integration Time	50
Profile	Helium KED
Ammonia	0
Helium	3
Hydrogen	0
Oxygen	0
RPa	0
RPq	0.25

Table S7. ^{57}Fe ICP-MS Method

Parameter	Operating Condition
Sweeps/Reading	5
Readings/Replicate	5
Replicates	5
Internal Standard ^{45}Sc	
Scan Mode	MS/MS
MCA Channels	1
IGM	Focusing
Dwell Time/ AMU	2
Integration Time	50
Profile	Standard
Ammonia	0
Helium	0
Hydrogen	0
Oxygen	0
RPa	0
RPq	0.25
^{57}Fe	
Scan Mode	MS/MS
MCA Channels	1
IGM	Focusing
Dwell Time/ AMU	2
Integration Time	50
Profile	Helium KED
Ammonia	0
Helium	3
Hydrogen	0
Oxygen	0
RPa	0
RPq	0.25

Table S8. ^{208}Pb ICP-MS Method

Parameter	Operating Condition
Sweeps/Reading	5
Readings/Replicate	5
Replicates	5
Internal Standard ^{45}Sc	
Scan Mode	MS/MS
MCA Channels	1
IGM	Focusing
Dwell Time/ AMU	2
Integration Time	50
Profile	Standard
Ammonia	0
Helium	0
Hydrogen	0
Oxygen	0
RPa	0
RPq	0.25
^{208}Pb	
Scan Mode	MS/MS
MCA Channels	1
IGM	Focusing
Dwell Time/ AMU	2
Integration Time	50
Profile	Standard
Ammonia	0
Helium	0
Hydrogen	0
Oxygen	0
RPa	0
RPq	0.25

Table S9. ^{88}Sr ICP-MS Method

Parameter	Operating Condition
Sweeps/Reading	
Readings/Replicate	
Replicates	
Internal Standard ^{45}Sc	
Scan Mode	MS/MS
MCA Channels	1
IGM	Focusing
Dwell Time/ AMU	2
Integration Time	50
Profile	Standard
Ammonia	0
Helium	0
Hydrogen	0
Oxygen	0
RPa	0
RPq	0.25
^{88}Sr	
Scan Mode	MS/MS
MCA Channels	1
IGM	Focusing
Dwell Time/ AMU	2
Integration Time	50
Profile	
Ammonia	0
Helium	0.8
Hydrogen	0
Oxygen	0
RPa	
RPq	

Table S10. BLADE MWDS Settings for Bone Method

**Stage 1**	
Temperature (°C)	220
Pressure (psi)	700
Stirring	Medium
Ramp Time	5:00
Hold Time	5:00
**Vent Pressure (PSI)**	
1	225
2	250
3	275
4	325
5	750
**Vent Count**	
1	2
2	2
3	2
4	2
5	∞
**Sensors**	
Temperature (°C)	42.2
I/O Board Temperature (°C)	39.7
AC Board Temperature (°C)	33.9
Pressure (psi)	0.0
Line Voltage (V)	120.0
Line Frequency (Hz)	60
